# Predictive and tempo-flexible synchronization to a visual metronome in monkeys

**DOI:** 10.1038/s41598-017-06417-3

**Published:** 2017-07-21

**Authors:** Ryuji Takeya, Masashi Kameda, Aniruddh D. Patel, Masaki Tanaka

**Affiliations:** 1 Department of Physiology, Hokkaido University School of Medicine, Sapporo, 060-8638 Japan; 2 Department of Psychology, Tufts University, Medford, MA 02155 USA; 3 Azrieli Program in Brain, Mind, and Consciousness, Canadian Institute for Advanced Research (CIFAR), Toronto, ON Canada

## Abstract

Predictive and tempo-flexible synchronization to an auditory beat is a fundamental component of human music. To date, only certain vocal learning species show this behaviour spontaneously. Prior research training macaques (vocal non-learners) to tap to an auditory or visual metronome found their movements to be largely reactive, not predictive. Does this reflect the lack of capacity for predictive synchronization in monkeys, or lack of motivation to exhibit this behaviour? To discriminate these possibilities, we trained monkeys to make synchronized eye movements to a visual metronome. We found that monkeys could generate predictive saccades synchronized to periodic visual stimuli when an immediate reward was given for every predictive movement. This behaviour generalized to novel tempi, and the monkeys could maintain the tempo internally. Furthermore, monkeys could flexibly switch from predictive to reactive saccades when a reward was given for each reactive response. In contrast, when humans were asked to make a sequence of reactive saccades to a visual metronome, they often unintentionally generated predictive movements. These results suggest that even vocal non-learners may have the capacity for predictive and tempo-flexible synchronization to a beat, but that only certain vocal learning species are intrinsically motivated to do it.

## Introduction

When processing certain temporal patterns humans often perceive a “beat” or underlying metronome-like periodicity^1^. It is common for humans to spontaneously synchronize rhythmic movements to this beat, a response that is fundamental to dance in every known culture^2^. Two key properties of these movements are that they are predictive and tempo-flexible. “Predictive” means that movements anticipate the beat rather than react to it. For example, when tapping with a metronome, humans align their taps very closely in time with metronome events, often tapping slightly before each event. “Tempo-flexible” means that this sort of predictive synchronization generalizes across a broad range of tempi^3^.

To date, spontaneous predictive and tempo-flexible synchronization to an auditory beat has only been demonstrated in humans and parrots^4, 5^, while predictive synchronization has also been reported in a chimpanzee^6^, a bonobo^7^, macaque monkeys^8^, and Asian elephants^5^ under limited conditions (e.g., for a specific tempo, in the presence of visual cues, or under social situations). Because humans and parrots share the rare ability to imitate complex sounds^9^, the evolution of vocal learning might develop auditory-motor connections in the brain that are necessary for predictive and tempo-flexible synchronization^10–12^. However, a challenge to this “vocal learning and rhythmic synchronization” hypothesis is the finding that a putative vocal non-learning animal, the California sea lion, was able to acquire the ability through reward-based training^13, 14^. This raises the possibility that the capacity for predictive and tempo-flexible synchronization is widespread in animal brains including vocal non-learners, but that only certain vocal learning species are intrinsically motivated to engage in this behaviour. This idea can be referred to as the “intrinsic reward and rhythmic synchronization” hypothesis.

One way to distinguish between the vocal learning hypothesis and the intrinsic reward hypothesis is to test animals that are definitely vocal non-learners without close vocal-learning relatives. Macaque monkeys are ideal animals in this regard, because they are known vocal non-learners without close vocal-learning relatives and are capable of learning complex sensorimotor tasks including those requiring temporal prediction^15–24^. The first study of macaque synchronization to a beat used both auditory and visual metronomes at several different tempi and trained the animals to tap in time with a series of 4 metronome events and then to continue with 3 self-timed taps at the same tempo^25^. Juice rewards were used after each trial to motivate learning. The animals were able to learn the task, but taps lagged metronome events by about 300 ms. This latency was faster than the monkeys’ reaction time to randomly-timed events, but was still quite different from humans tested on the same task, who had latencies near 0 ms. Although subsequent work in the same lab attempted to reduce the latency of monkey tapping by requiring smaller latencies for reward, the shortest tapping latency was ~100 ms, with taps always following metronome events rather than preceding them, on average^26^. These results were inconsistent with the intrinsic reward hypothesis, which predicts that monkeys should be capable of synchronizing to metronome with near zero (or even negative) mean asynchronies even for untrained tempi. Instead, these observations have led to the hypothesis that the ability of beat-based timing is unique to humans among primates, while humans and monkeys fully share the ability of single-interval timing (“gradual audiomotor evolution” hypothesis)^26^.

The current study tests synchronization to a beat using methods somewhat different from the previous work. While we also trained monkeys on a synchronization task, we used voluntary eye movements rather than taps as a motor behaviour, and a spatialized visual metronome rather than a stationary flashing metronome. Predictive saccades to spatially distinct locations are ecologically natural behaviours in monkeys, whereas predictive hand movements to a spatially stationary stimulus may not be natural for these animals. Our study also differed from previous work in terms of the structure of the reward schedule. In the series of previous studies^25–27^ monkeys received a single reward at the end of each trial, while in the current study they obtained a reward for *each synchronized movement* after the first few movements. Once monkeys had been trained to synchronize at a specific set of metronome tempi, they were tested for generalization to new, untrained tempi.

Since our study focuses on motor synchronization to a *visual* beat, it is not a direct test of the hypotheses concerned with auditory-motor synchronization, such as the vocal learning hypothesis^10^ and the gradual audiomotor evolution hypothesis^26^. However, our study is a direct test of the intrinsic reward hypothesis (which predicts that even vocal non-leaners have the capacity for predictive and tempo-flexible synchronization), and is a first step in directly testing the aforementioned hypotheses concerned specifically with auditory beats. Our results show that predictive and tempo-flexible synchronization to a beat is possible for monkeys in the visual modality, and provide a new method that could be adapted for the study of auditory synchronization in monkeys.

## Results

**Figure 1:**
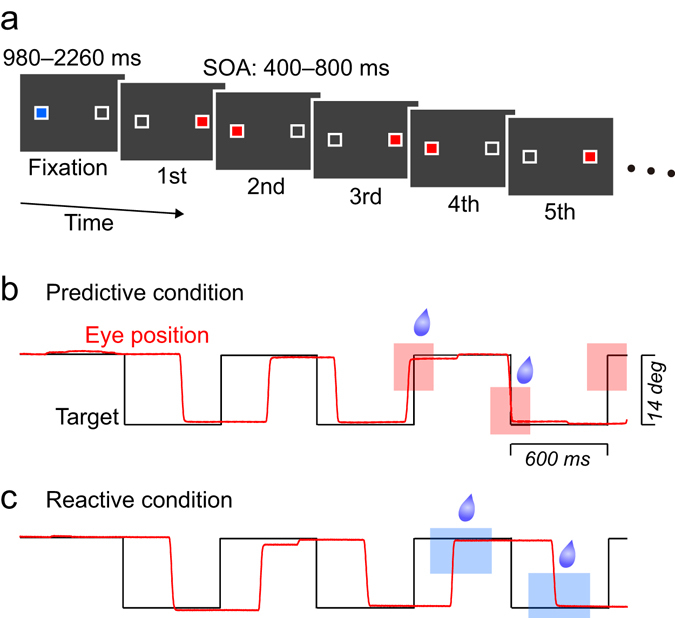
Behavioural paradigm. (**a**) Two unfilled white square landmarks were presented horizontally (14° apart) throughout the trial. A fixation point (blue rectangle) appeared at either landmark location. After a random fixation period, a saccade target (red or green rectangle) was presented at the other landmark. The target alternated with a constant stimulus onset asynchrony (SOA) that was randomly chosen from 400–800 ms (100 ms step; 300, 400, 800 and 900 ms during training sessions, see Methods) in each trial. (**b**) Data from a sample trial with a 600-ms SOA. In the predictive condition, monkeys were rewarded for every predictive saccade that was generated within the specific temporal window (±20% SOA from target onset, pink rectangles). (**c**) In the reactive condition, animals were rewarded for every reactive saccade that was generated >100 ms from the target onset until 20% SOA before the next target (blue rectangles). Two conditions were presented in separate experimental sessions. Red and green targets were used for the predictive and the reactive conditions, respectively.

**Figure 2:**
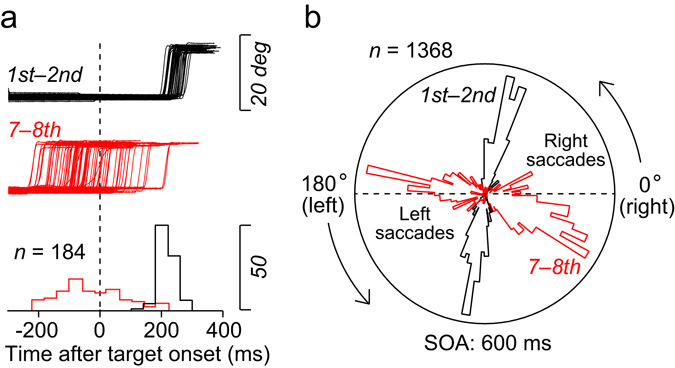
Reactive and predictive saccades in representative experiments. (**a**) Eye position traces of the 1st–2nd (black) and 7–8th (red) rightward saccades for trials with a 600-ms SOA in a single experimental session. Data are aligned with the target onset (vertical dashed line), and lower histograms summarize saccade latencies. Note that while the initial two saccades in the sequence were reactive, the later saccades were often synchronized with (or even preceded) target appearance. (**b**) Circular histogram of saccade timing for 5 experimental sessions in monkey K. 0° and 180° indicate the timing of right and left target onset, respectively. Each cluster of the histogram is normalized for the peak value.

Three Japanese macaques were trained to generate sequential saccades to a visual target presented at two landmark locations (Fig. 1a). During the task, the stimulus alternated at a fixed interval (stimulus onset asynchrony; SOA) that was chosen randomly from 400–800 ms in each trial (SOAs differed in steps of 100 ms, yielding 5 possible SOAs). To facilitate predictive responses, saccades generated within ±20% SOA from target appearance were reinforced immediately with a liquid reward (e.g., for a 500-ms SOA, the reinforcement window extended from 100 ms before to 100 ms after the target onset, see Fig. 1b, predictive condition). Figure 2a plots the traces of eye position and the associated latency histograms for the 1st–2nd (black) and the 7–8th (red) rightward saccades in the 600-ms SOA sequence in a single experimental session of monkey K. While the initial two saccades were reactive and had a mean latency of 254 ± 42 ms (SD), the later saccades were predictive and occurred around the time of target onset (mean ± SD, −34 ± 104 ms). These latency values were statistically different (unpaired *t*-test, *t*_(182)_ = 28.4, *p* < 10^−61^). Figure 2b shows a circular histogram of saccade latency obtained from 5 experimental sessions with the same monkey, comparing the early and late saccades in the sequence in trials with a 600-ms SOA. For both saccade directions, the latency of the 1st–2nd saccades significantly differed from that of the 7–8th saccades (Watson-Williams test^28^, *F*_(1,8)_ = 1131.6, *p* < 10^−10^). Thus, the monkey could adjust saccade timing to a periodic stimulus after some repetition.

### Generalization of synchronized saccades to untrained rhythms and locations

Since the first monkey (K) was extensively trained to generate predictive saccades for the SOAs of 300–900 ms (Methods), one might argue that the animal learned sequential saccades with specific rhythms or inter-saccadic intervals (ISIs). To examine whether the animals could generalize synchronized saccades to novel SOAs, two monkeys (X and J) were initially trained in trials with SOAs of 300, 400, 800 and 900 ms for several weeks. Then, the animals were presented with a block of target sequence with SOAs of 400, 500, 600, 700 and 800 ms during 5 test sessions. Before these sessions, these monkeys had never been exposed to the target sequences with SOAs of 500, 600 and 700 ms.

**Figure 3:**
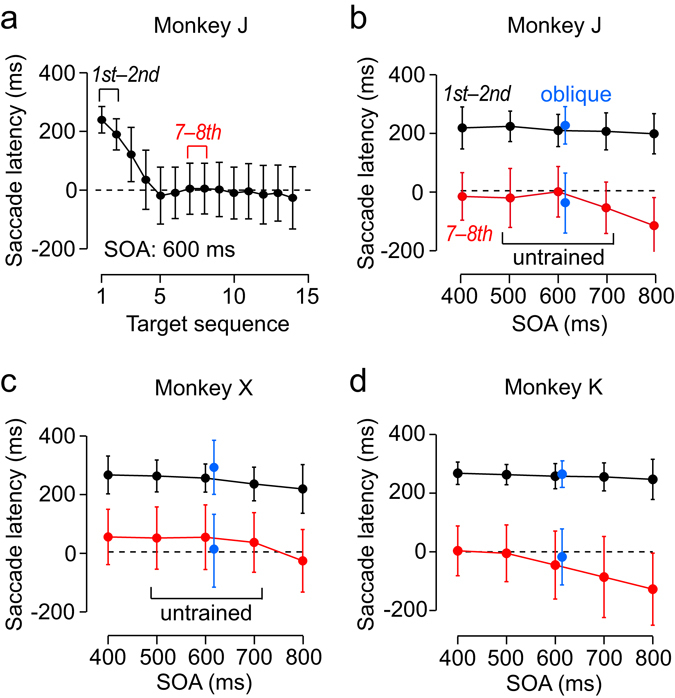
Generalization of predictive synchronization to novel SOAs and target locations. (**a**) Mean saccade latency in the predictive condition as a function of target sequence in monkey J. Each data point represents the mean of 409 saccades from five experimental sessions and the error bar indicates trial-by-trial variation (±SD). (**b**) Mean saccade latencies for different SOAs in monkey J. Although the animal was trained only for shorter and longer SOAs (300, 400, 800 and 900 ms), he could make synchronized saccades for novel SOAs of 500–700 ms. Generalization also occurred for oblique targets (blue symbols). Error bars indicate ± SD of individual trials pooled across 5 experiments. Each data point denotes the mean of 732–818 saccades. (**c**,**d**) Data from two other monkeys. Note that monkey K had been trained for all SOAs and target locations, but monkey X had never been presented with SOAs of 500–700 ms or oblique targets before the test sessions. Corresponding circular histograms are shown in Supplementary Fig. S1. Data from 5 individual sessions in monkeys J and X are shown in Supplementary Fig. S2.

Figure 3a plots the mean and SD (inter-trial variability) of saccade latency as a function of position in the target sequence with an untrained 600-ms SOA in monkey J. Because trials with different SOAs were intermingled randomly in a block, the initial few saccades were reactive and had longer latency. However, the animal rapidly reduced saccade latency and synchronized with the stimulus sequence of an untrained SOA. Figure 3b–d compare the mean (±SD) latencies of the 1st–2nd and 7–8th saccades for different SOAs (Supplementary Fig. S1 shows corresponding circular histograms). Importantly, while monkeys J and X had never experienced the trials with SOAs of 500–700 ms, the later saccades in the sequence had shorter latencies than the earlier saccades for all SOAs (Fig. 3b and c). In other words, trained and untrained SOAs evoked similar qualitative patterns of response. This was also evident even in the first of 5 experimental sessions for these two animals (Supplementary Fig. S2). A two-way analysis of variance (ANOVA) showed significant main effects on saccade latency for all animals (target sequence, *F*_(1,40)_ = 866.9, 1437.8, 1496.8 for monkeys J, X and K, respectively, *p*s < 10^−5^; SOA, *F*_(4,40)_ = 10.39, 13.44 and 14.1, respectively, *p*s < 0.05). Although only the data from monkey K showed a significant interaction (*F*_(4,40)_ = 5.26, *p* < 0.05), post hoc multiple comparisons revealed that the 7–8th saccades had shorter latency than the 1–2nd saccades for all SOAs (*p*s < 10^−5^). We also conducted a three-way ANOVA (subjects × SOA × target sequence) to explore individual differences. The results showed that all main factors were statistically significant (subjects, *F*_(1,120)_ = 31.5, *p* < 10^−10^; target sequence, *F*_(1,120)_ = 4644.7, *p* < 10^−90^; SOA, *F*_(4,120)_ = 39.3, *p* < 10^−20^), with a significant three-way interaction (*F*_(8,120)_ = 2.26, *p* < 0.05) that reflected the interaction of SOA and target sequence in one monkey (K). Taken together, these results indicate that the animals could generate predictive saccades synchronized with metronomic visual stimuli at novel tempi.

We also asked whether the animals could generate synchronized saccades to novel target locations. Even when the landmark locations were shifted vertically in a fraction of trials with a 600-ms SOA, we again found a decrement of latency of the 7–8th saccades compared to the 1st–2nd saccades in all monkeys (Fig. 3b–d, blue symbols, monkey J: *t*_(4)_ = 11.1, *p* < 10^−4^; monkey X: *t*_(4)_ = 15.3, *p* < 10^−4^; monkey K: *t*_(4)_ = 38.1, *p* < 10^−5^). A two-way ANOVA (subjects × target sequence) showed a significant main effect of target sequence (*F*_(1,24)_ = 188.3, *p* < 10^−12^) with no interaction (*F*_(1,24)_ = 0.6, *p* = 0.55). Thus, the animals could also generalize synchronized saccades to untrained target locations.

### Maintenance of internal rhythms

So far, we have shown that monkeys can generate predictive synchronized saccades to periodic visual stimuli. The generalization to novel metronome tempi suggests that the animals are able to extract the temporal structure or “beat” of the visual stimulus sequence and match the movement timing with this beat — in other words, they can entrain to a visual metronome in a predictive and tempo-flexible way. To better understand the underlying mechanisms, we next examined whether the animals could maintain isochronous rhythms in the absence of temporal “error” or the time discrepancy between movements and target appearance. In the “error-clamp” trials, targets were presented with a certain SOA during the initial half of the trial (4 sec), and then the target was presented in the wake of each predictive saccade during the latter half of the trial (i.e., the target appeared when the monkeys looked at the landmark location). The error-clamp (or error-free) manipulation was different from the “continuation” condition in previous studies in that the target was presented for each movement irrespective of its timing.

**Figure 4:**
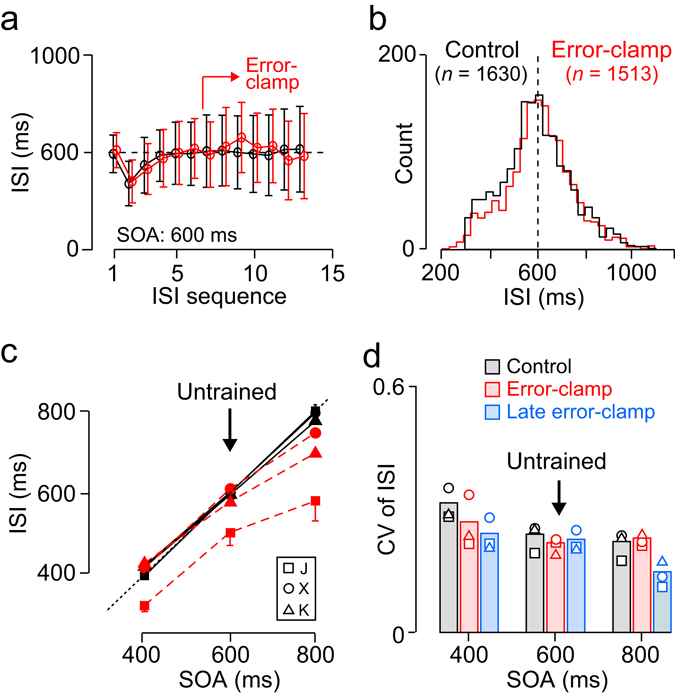
Entrainment to periodic stimuli. (**a**) Inter-saccadic intervals (ISIs) are plotted as a function of saccade sequence for 5 experimental sessions in monkey X. In the error-clamp trials (red), the target appeared when the monkey looked at the target location (≥7th saccade in the sequence). Error bars indicate ± SD (inter-trial variation). (**b**) Distributions of ISIs for the 7–12th saccades (clamp period) in trials with (red) and without (black) error-clamp. (**c**) The ISIs as a function of SOA in the error-clamp (red dashed lines) and non-clamp (control) conditions (black solid lines) for different animals. Error bars indicate SDs of 5 experimental sessions and are shown only for some data points. (**d**) Coefficient of variation (CV) of ISI for different SOAs. Each data point shows the mean of 5 sessions for each monkey. Blue bars indicate the data during the late part (>2000 ms) of error-clamp condition.

Figure 4a plots the inter-saccadic interval (ISI) as a function of target sequence in trials with a 600-ms SOA in monkey X. The time courses of the ISI were quite similar between the trials with and without the error-clamp manipulation (red and black points, respectively). The distributions of the ISI for the 7–12th saccades in the sequence pooled across 5 experiments were also similar between the conditions, although a statistical test between the large samples detected a small but significant mean difference (Fig. 4b; mean ± SD, 611 ± 166 ms and 602 ± 158 ms for the error-clamp and control conditions, respectively; unpaired t-test, *t*_(3141)_ = 2.8, *p* < 0.01). Figure 4c summarizes the data from all three monkeys for different SOAs. Two-way ANOVAs (SOA × clamp condition) for the ISI showed significant main effects (SOA, *F*_(2,24)_ = 435.9, 861.2, 397.1 for monkeys J, X and K, respectively, *p*s < 10^−18^; clamp condition, *F*_(1,24)_ = 201.2, 4.6, 9.8, respectively, *p*s < 0.05) and interactions for all monkeys (J: *F*_(2, 24)_ = 24.9, X: *F*_(2, 24)_ = 6.2, K: *F*_(2, 24)_ = 8.6, *p*s < 0.01). Post hoc multiple comparisons for the error-clamp data indicated that the ISIs steadily increased as the SOA increased in all animals (*p*s < 0.05). Moreover, the ISIs for 800-ms SOA tended to be shorter in the absence of temporal feedback in all animals (unpaired t-test, J: *t*_(8)_ = 10.0, X: *t*_(8)_ = 3.3, K: *t*_(8)_ = 5.1, *p*s < 0.01), while significant changes in ISIs between the control and error-clamp conditions for 400 and 600-ms SOAs were found only in one monkey (J, *p*s < 10^−3^). Thus, the animals could entrain to periodic visual stimuli, while the internal rhythm appeared to be shorter when the SOA was relatively long. The latter result might be due to the combination of general facilitation of periodic saccades during the error-clamp manipulation and the bias of temporal estimation toward the mean of SOAs^29^. By this we mean that for longer SOAs in the control condition, the animals might delay eye movements in order to compensate for the faster internal tempo since sensory feedback for timing was available. In this case, the variability of ISI during the error-clamp condition would be smaller than the control condition, because in the control condition saccade timing would depend both on the sensory feedback and internal rhythm. However, two-way ANOVAs (clamp condition × SOA) for the coefficient of variation (CV) of ISI (Fig. 4d, grey and red bars) detected a significant clamp effect only in one monkey (K: *F*_(1,4)_ = 4.8, *p* < 0.05), indicating that the variation of saccade timing was similar in both conditions. The effect of SOA was also significant in two monkeys (J: *F*_(2, 24)_ = 20.3, *p* < 10^−5^; X: *F*_(2, 24)_ = 35.5, *p* < 10^−7^), and post hoc multiple comparisons indicated that the CVs were greater for the 400-ms SOA than the other SOAs in these animals (*p*s < 0.05).

It is known that when humans tap in synchrony with an auditory metronome, the duration of successive inter-tap-intervals is often negatively correlated (i.e., a longer interval is often followed by a shorter one), which is often taken as evidence of a rapid error-correction mechanism^30^. In contrast, in human tapping with a stationary visual metronome, successive inter-tap-interval durations are often positively correlated, indicating a slow drift in inter-tap-interval duration^30^. To further elucidate how monkeys maintained internal rhythm, we computed Pearson’s correlation coefficient between successive ISIs for the untrained 600-ms SOA (Supplementary Fig. S3a and b). Interestingly, in our monkeys successive ISIs were negatively correlated throughout the trials under the non-clamp control condition (solid lines), indicating that timing of each saccade was modified depending on the temporal error of the preceding saccade. Even after the onset of the error-clamp manipulation, the negative correlation persisted for a few saccades, while later correlation coefficients rapidly became positive (circles with dashed lines). These results indicate that the animals adjusted saccade timing to the remembered external rhythm during the initial few saccades in the error-clamp condition, while the internal rhythm steadily deviated from the external rhythm in the later part of the trial.

We also performed an analysis to verify that monkeys adjusted saccade timing in a manner sensitive to previous saccades rather than resetting internal timing for every saccade^31^. Supplementary Fig. S3c plots the SDs of saccade timing relative to the start of the error-clamp manipulation (red), compared to the results of a simulation based on the “single-interval model” (black). For this simulation, the variation of timing of each saccade was computed by shuffling ISIs across trials (1000 repeats, see Methods). The data show that variation of saccade timing was significantly smaller than the prediction of the model during the initial part of the error-clamp period, indicating that monkeys determined saccade timing in a manner sensitive to sequence structure until a few seconds following the start of the error-clamp manipulation.

The results in Supplementary Fig. S3 indicate that even in the absence of sensory feedback, saccade timing was regulated based on the timing of the preceding saccade during the initial part of error-clamp condition; however, during the later part of the error-clamp condition such regulation did not occur and saccade timing might solely depend on the internal rhythm. We therefore again expected that the CVs of ISIs during the late part of error-clamp condition (Fig. 4d, blue bars) would be smaller than those of control (grey bars), because saccade timing was solely under internal control during that period. Two-way ANOVAs (clamp condition × SOA) detected a significant main effect of error-clamp for all monkeys (J: *F*_(1,24)_ = 15.6, X: *F*_(1,24)_ = 10.1, K: *F*_(1,24)_ = 15.5, *p*s < 0.01) and a significant effect of SOA and interaction for two monkeys (SOA, J: *F*_(2,24)_ = 34,0, X: *F*_(2,24)_ = 75,6, *p*s < 10^−7^; interaction, J: *F*_(2,24)_ = 3, 6, X: *F*_(2,24)_ = 7.8, *p*s < 0.05). These results were in line with the idea that monkeys can entrain to periodic visual stimuli and can also generate movements using an internal rhythm derived from these stimuli.

The error-clamp data described above strongly suggest that the animals entrained their internal rhythm to a given stimulus sequence. However, because the reward was delivered for each predictive saccade, it might be still possible that the animals reset their internal timing for a reward and measured single time intervals for each saccade. However, we found that once monkeys were motivated to generate phase-leading synchronized saccades, an immediate reward for every predictive movement was no longer necessary. Supplementary Fig. S4 shows that all trained animals generated predictive synchronized saccades even when a reward was given for every third movement. A two-way ANOVA (reward schedule × target sequence) for the 1st–2nd and the 7–8th saccade latencies detected a significant effect of target sequence (*F*_(1, 12)_ = 184.0, *p* < 10^−7^), but no effect of reward schedule or interaction (*p*s > 0.78). Thus, although the immediate reward might be necessary for initial training, the feedback of reward on each saccade was no longer necessary for phase-leading synchronization in trained monkeys.

### Flexible switching of behavioural strategy in monkeys and humans

In the preceding sections, we have shown that monkeys are capable of generating predictive and tempo-flexible movements synchronized to visual metronomes when each predictive response is reinforced by an immediate reward. Because previous studies reported that monkeys generated only reactive tapping^25^ or saccades^32^ to metronomic visual stimuli, we next asked how flexibly the animals can switch between predictive and reactive responses to the periodic stimuli.

**Figure 5:**
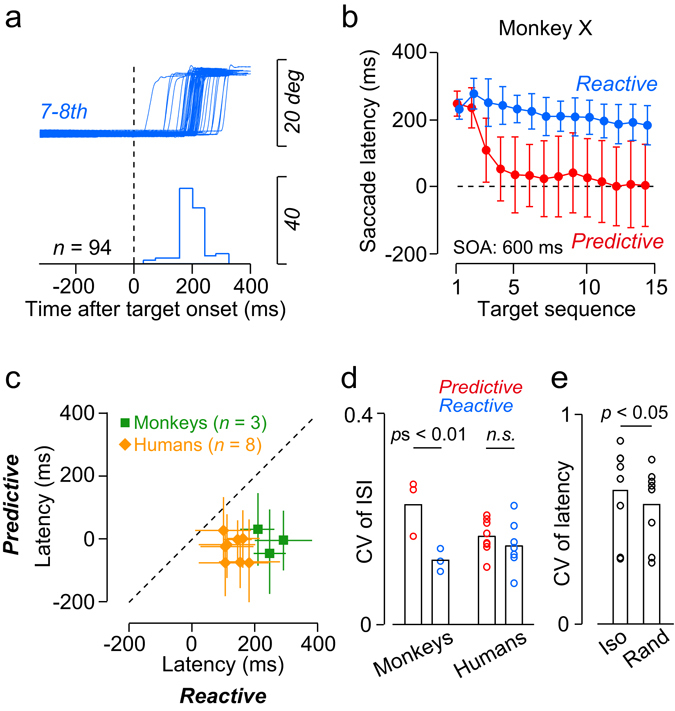
Flexible switching of behavioural strategy. (**a**) Data from a single experimental session in the reactive condition for monkey K. Traces of eye position are aligned on the 7–8th target onset (vertical dashed line) in trials with a 600-ms SOA. The animal was rewarded for every reactive saccade (Fig. 1c, see Methods). (**b**) Means (±SDs) of saccade latencies as a function of target sequence in the reactive (blue) and predictive (red) conditions. Data for each condition were obtained from 5 experimental sessions. (**c**) Comparison of the means of the 5–12th saccade latencies between the conditions in trials with a 600-ms SOA. Symbols with different colours indicate different species. Each data point indicates the mean (±SD) of individual trials in all experimental sessions for each subject. Note that saccade latency in the reactive condition was shorter for humans than monkeys. (**d**) Comparison of CVs of inter-saccadic intervals (ISIs) measured for different conditions and species. For all cases, CVs were measured for the 4–11th ISIs in the sequence. Each bar indicates the mean of different subjects. (**e**) Comparison of CVs of saccade latencies between the reactive condition with an isochronous stimulus sequence (600-ms SOA) and the random SOA condition for humans.

Figure 5a shows the traces of the 7–8th rightward saccades in a single experimental session when a reward was delivered for each reactive saccade (cf. Fig. 1c, reactive condition). Unlike the traces in Fig. 2a, where the monkey switched from reactive to predictive saccades during each trial, the same monkey (K) generated only reactive saccades in most trials. As in the case of the predictive condition, two monkeys (X and J) were trained for the reactive condition with SOAs of only 300, 400, 800 and 900 ms for a few weeks, and then were presented with a block of trials with SOAs of 400, 500, 600, 700, and 800 ms in 5 test sessions. Figure 5b plots the mean (±SD) saccade latency as a function of target sequence for a 600-ms SOA in both reward conditions in monkey X. In contrast to the predictive condition (red), saccade latencies in the reactive condition (blue) did not alter greatly as the sequence progressed. When we compared the means of the 7–8th saccade latencies between the reward conditions, all animals showed significant difference (unpaired t-test, *t*_(4)_ = 16.1, 19.3 and 14.3 for monkeys X, J and K, respectively, *p*s < 10^−6^). Thus, our monkeys could switch from predictive to reactive saccades to obtain rewards.

As noted in the Introduction, the tendency to spontaneously generate predictive and tempo-flexible movements synchronized to a beat might differ between vocal learners and non-learners^33^. Therefore, we also examined sequential saccades in humans (vocal learners) for comparison with monkeys (vocal non-learners). Participants were instructed to make a series of predictive or reactive saccades depending on target colour (see Methods). Orange diamonds in Fig. 5c compare the means of the 5–12th saccade latencies in the sequence between the two conditions, showing a clear reduction of latency in the predictive condition. A two-way ANOVA (species × reward condition) for saccade latency showed significant main effects (species, *F*_(1,18)_ = 16.3, *p* < 0.001; reward condition, *F*_(1,18)_ = 138.2, *p* < 10^−9^) and interaction between them (*F*_(1,18)_ = 4.54, *p* < 0.05). Post hoc multiple comparisons indicated that saccade latency was shorter for humans than monkeys only in the reactive condition (135 ± 31 ms versus 256 ± 41 ms unpaired t-test, *t*_(9)_ = 5.42, *p* < 0.001), although saccade latency is generally shorter in monkeys than humans in the literature^34, 35^. This could be because humans *spontaneously* entrained to the periodic rhythm and sometimes generated predictive saccades even in the reactive condition. In fact, the proportion of predictive saccades (latency < 100 ms) in the reactive condition was greater in humans than monkeys (27 ± 12% versus 4 ± 2%, Wilcoxon rank-sum test, *z* = 3.8, *p* < 0.01). Accordingly, the variance of saccade latency in the reactive condition was also greater in humans than monkeys. Figure 5d compares the CVs of inter-saccadic interval between conditions and species. Although the CV differed significantly between conditions in all monkeys (unpaired t-test, *t*_(4)_ = 3.5, 7.9, 5.8, for monkeys J, X and K, respectively, *p*s < 0.01), in humans it did not (paired t-test, *t*_(7)_ = 2.3, *p* = 0.06). Furthermore, when the human participants were presented with a non-periodic stimulus sequence with random SOAs (400–800 ms, 9 SOAs in 50 ms steps, equal probability), saccade latency significantly increased (135 ± 31 ms versus 167 ± 17 ms, paired t-test, *t*_(7)_ = 4.9, *p* < 0.01) and the CV of saccade latency decreased (Fig. 5e, *t*_(7)_ = 2.8, *p* < 0.05) as compared to the reactive condition with a fixed SOA. Moreover, the proportion of predictive saccades was reduced in the random SOA condition (16 ± 6%) in comparison with the reactive condition (Wilcoxon signed-rank test, *z* = 2.5, *p* < 0.05). These results indicate that the large variability of saccade latency in the isochronous reactive condition observed in humans (Fig. 5d) was unlikely to be related to the lower functional capacity of the oculomotor system; instead, humans tended to spontaneously predict and synchronize with periodic visual stimuli.

## Discussion

We found that macaque monkeys were capable of predictive synchronization of eye movements with metronomic visual stimuli when rewards were given for each predictive saccade. This predictive behaviour generalized to untrained metronome tempi and untrained saccade directions (Fig. 3). When rewards were given for reactive saccades, the same animals switched their behavioural strategy to generate only reactive saccades (Fig. 5). In contrast, when human participants were asked to make a sequence of reactive saccades to a metronomic visual stimulus, they often unintentionally generated predictive eye movements, making their saccade latency shorter and more variable than monkeys. These results show that the capacity for predictive and tempo-flexible synchronization to a visual metronome is present in monkeys, but (unlike for humans) is not intrinsically rewarded and has to be elicited through training with appropriate external rewards.

We found that periodic eye movements were maintained even when the timing of visual stimuli was linked to the animals’ rhythmic eye movements rather than being externally determined (Fig. 4), suggesting that the animals could internally maintain the rhythm of the preceding metronome. This is reminiscent of the findings of Zarco *et al*.^25^, who found that monkeys could continue to tap at the metronome tempo for a few cycles when the metronome was turned off. It is also reminiscent of other recent studies which have demonstrated that monkeys can maintain internal rhythms (i.e., in the absence of external rhythmic cues) that guide eye movements^20^ or spatial attention^27^.

However, our results contrast with previous studies in demonstrating *predictive* (phase-lead) and tempo-flexible synchronization to a visual metronome, including at novel tempi. This difference may stem from the difference in movement type (saccades versus tapping), or perhaps more importantly, from the fact that we reinforced each predictive movement with an immediate reward rather than waiting until the end of the sequence. We suspect that our reward schedule during training is a key factor, because in a prior study of saccades to visual targets that alternated rhythmically between two locations (with rewards given at the ends of trials), Fuchs (1967)^32^ found that monkeys did not move their eyes predictively. While immediate reward might be necessary to train monkeys to move predictively to rhythmic stimuli, we also found in this study that once animals were motivated to generate phase-lead synchronized movements, external feedback for every movement was no longer necessary (Supplementary Fig. S4).

A number of previous studies have reported predictive eye movements in monkeys. Like humans, monkeys can generate smooth pursuit eye movements for a sinusoidal motion trajectory with no phase lag^36, 37^. However, the underlying neural mechanisms for the control of continuous and discontinuous movements appear to be different^38^. Accumulating evidence shows that monkeys are also able generate predictive saccades in situations requiring them to monitor the passage of time and make discontinuous movements. For instance, monkeys can make self-initiated saccades during a specific time interval following a cue^16, 39, 40^. However, because these previous studies required long-term behavioural training, the animals might have learned specific temporal sequences of saccades in a given condition. In contrast, we found that monkeys could make predictive and *tempo-flexible* saccades for novel stimulus sequences, indicating that they can extract temporal information from visual metronomes and immediately adjust saccade timing.

An important question raised by our results when compared with previous studies^25, 41^ is whether similar neural mechanisms are used for synchronization of ocular versus hand movements with metronomic stimuli. One reason to suspect that these tasks may involve somewhat different brain networks (beyond primary motor control areas) is that humans spontaneously entrain rhythmic limb/trunk/head movements to a beat, but not eye movements. Indeed, although the areas of activation for synchronized tapping revealed by functional imaging largely overlap with those for synchronized saccades, some differences also exist. Specifically, while increased activity in the cortico-putaminal network has been consistently observed during synchronized tapping^42^, greater activation in the default mode network and the lateral cerebellum has been found during a sequence of predictive saccades as compared with reactive saccades^43^. Differences in neural activation might also reflect the fact that alternating saccades require temporal adjustment of multiple movements (i.e., those in opposite directions) while tapping depends solely on the timing of a single movement. Because both types of motor synchrony are common in human movement patterns (e.g., in dancing, swinging, typing), the possible difference between these conditions needs to be considered in future studies.

Another question raised by our findings is whether monkeys could learn to exhibit predictive movements to an *auditory* metronome. fMRI research in humans has shown distinct brain activation patterns for synchronization to discrete auditory versus visual metronomes for both ocular^43^ and tapping^44^ movements, beyond the obvious differences related to sensory processing. For example, predictive ocular synchronization to an auditory (but not visual) metronome is associated with increased activity in the inferior parietal lobe^43^, and tapping to metronomic beeps is associated with significantly stronger putaminal activation than tapping to metronomic flashes^44^. This latter neural difference may be relevant to the human tendency to exhibit substantially better tapping synchronization to discrete auditory than visual metronomes^3, 45^. Conversely, in monkeys performing a synchronized tapping task, sensory-responsive neurons in the medial premotor cortex showed a clear preference for visual over auditory stimuli, while stimulus-predicting neurons showed a bimodal response^46^. By taking advantage of the use of visual stimuli for behavioural training in monkeys, the present study clearly demonstrated that monkeys could learn to exhibit predictive and tempo-flexible synchronization, which was not observed in the previous studies^25, 32^. However, the strong preference for visual over auditory stimuli in monkey sensorimotor processing suggests that, unlike humans, monkeys may not show an auditory (relative to visual) advantage for ocular synchronization to a metronome. More generally, one may not assume from our results that predictive and tempo-flexible ocular (or tapping-based) synchronization to an auditory metronome is possible in monkeys. This ability remains to be tested.

Extensions of the current paradigm (in which every predictive movement is rewarded) to auditory synchronization tasks are worth pursuing because predictive and tempo-flexible synchronization to an auditory beat in monkeys would refute the “gradual audiomotor evolution hypothesis”^25^ and the “vocal learning and rhythmic synchronization hypothesis”^10^ in their current forms. Furthermore, it would shift the research focus to understanding why certain species *spontaneously* engage in this type of synchronization to an auditory beat (such humans and parrots) while others do not (such as sea lions or monkeys). The “intrinsic reward and rhythmic synchronization hypothesis” posits that many species (including vocal non-learners) have the capacity for predictive and tempo-flexible synchronization to a beat (whether visual or auditory), but that only certain vocal learning species are intrinsically motivated to do it. While the reasons for this intrinsic motivation have yet to be understood, the hypothesis is supported by the findings that the reward system in the basal ganglia exhibits enhanced activity when human subjects generate predictive movements synchronized to an auditory beat^47, 48^. By adapting the current methods to study synchronization to an auditory beat, one can test the opposing predictions of the intrinsic reward hypothesis versus the vocal learning hypothesis and the gradual audiomotor evolution hypothesis. In addition, the present behavioural paradigm offers the opportunity to explore the underlying neural mechanisms of predictive and tempo-flexible synchronization to a beat in monkeys.

## Methods

### Procedures of animal experiments

Two male and one female Japanese monkeys (*Macaca fuscata*, 6–9 kg, 4–8 years old) were used. For the experiments summarized in Supplementary Fig. S4, two additional animals were also recruited (one male and one female, 8 and 6 kg, both 7 years old). All experimental protocols were approved in advance by the Animal Care and Use Committee of Hokkaido University. The procedures for animal preparation are described in detail elsewhere^49^. Briefly, a pair of head holders was implanted to the skull using titanium screws and dental acrylic under general isoflurane anaesthesia. A coil of stainless steel wire was implanted under the conjunctiva to record eye movements. During the subsequent training and experimental sessions, the monkey’s head was secured to the primate chair, and horizontal and vertical eye position were recorded using the search coil technique (MEL-25, Enzanshi Kogyo).

All experiments were controlled by a Windows-based real-time stimulus presentation and data acquisition system (TEMPO, Reflective Computing) that updated all stimulus events at 200 Hz and acquired the data at 1 kHz. Visual stimuli were presented on a 27-inch liquid crystal display (XL2720Z, BenQ, refresh rate: 144 Hz) that was positioned 40 cm from the eyes and subtended 73° × 46° of visual angle. Throughout the experiment, two landmarks (white unfilled 1° squares) were presented ±7° horizontally (Fig. 1a) on the black background. In a fraction of trials, the landmark location was shifted 4° vertically (oblique condition). The fixation point (blue) and saccade target (red or green) was always presented within the landmark.

Animals were trained to follow the target with their eyes. Each trial started with the appearance of the fixation target (blue square, 10.9 cd/m^2^) at either landmark location. After a random (980–2260 ms) fixation period, the saccade target (red or green) appeared on the opposite side. The target was alternately presented at the landmark locations with a constant SOA (ranged from 300–900 ms, 100 ms steps, 7 SOAs) for 8000–8400 ms (i.e., 10–28 targets depending on the SOA). Each target was illuminated throughout SOA, until the target at the other position appeared. The trial was aborted if the 1st or 2nd saccade in the sequence was predictive (generated within 100 ms or 20% SOA of the target onset), or the inter-saccadic interval (ISI) deviated from the range of 25–200% SOA, or eyes deviated 3.5° vertically from the target locations.

Each animal performed the task in two different reward conditions. Before each training or experimental session, we properly regulated water supply to make the animals motivated to perform the task. In the predictive condition (Fig. 1b), the saccade target was red (33.9 cd/m^2^) and each predictive saccade (generated within ±20% SOA from the target onset) for the 4th or later target was reinforced with a drop of liquid reward. Because the initial fixation period and SOA varied from trial to trial, monkeys were unable to predict timing of the initial two stimuli in the sequence. To prevent anticipatory saccades at the start of a trial, trials with early saccades (<100 ms) for the initial two targets were aborted immediately and repeated later in the block of trials. In the reactive condition (Fig. 1c), we presented a green target (104 cd/m^2^) and rewarded each reactive saccade that was generated between 100 ms after target onset and 20% SOA before the next stimulus for the 4th or later target. For both conditions, the amount of reward for each saccade was adjusted so that the total amount of reward in each trial was roughly the same across SOAs. Animals were initially trained in the predictive condition for several weeks until they generated predictive saccades for more than half of target appearance. During the initial phase of the training, we delivered a small amount of reward for each reactive saccade to motivate monkeys to look at the target. We also delivered a large amount of reward for occasional predictive saccades and gradually altered the temporal requirement of saccades to obtain reward. To examine whether the animals could generate synchronized saccades to the stimulus sequence of untrained SOAs, two monkeys (X and J) were presented with trials of SOAs of only 300, 400, 800 and 900 ms during the training sessions. Then, the animals performed 5 test sessions that consisted of trials with SOAs of 400, 500, 600, 700 and 800 ms. The remaining monkey (K) was extensively trained for all SOA conditions.

In the predictive condition, we also presented two other stimulus conditions. Firstly, to examine whether monkeys could generate synchronized saccades to untrained target locations, the landmarks were located obliquely (4° above or below the horizontal meridian) in 12.5% trials during the 5 test sessions. Secondly, to examine whether the animals could entrain to the stimulus sequence and keep the internal rhythm in the predictive condition, we introduced an “error-clamp” condition in which the target appeared at the time of a predictive saccade. These trials were presented during both the training (400- and 800-ms SOAs) and test sessions (400-, 600- and 800-ms SOAs). During the error-clamp condition we delivered an immediate reward for all saccades that were generated >4 s following the first target onset.

### Procedures of human experiments

Eight healthy individuals (22–28 years old, 3 females), including two authors participated in the experiments. All had normal vision. The experiments in humans were evaluated and approved by the Ethics Committee of Hokkaido University Graduate School of Medicine, and were conducted in accordance with the Declaration of Helsinki. Written informed consent was obtained from each participant.

Participants were seated on a chair in front of a computer monitor. Their heads were restrained by a chinrest and a head-holding device. Their right eye was positioned in line with the centre of the screen that was located 40 cm from the eye. Horizontal position of the right eye was recorded using an infrared eye tracker (Takei Co, Eye movement monitor, DC–33 Hz, 24 dB/oct).

Subjects performed three sequential saccade tasks: tasks with a fixed 600-ms SOA under instruction of either predictive or reactive saccades, and a task with random SOAs (ranged from 400–800 ms, 9 SOAs in 50 ms steps, uniform distribution). As in the animal experiments, target colour was red for the predictive condition and green for the reactive and the random conditions. In all conditions, each trial started when subjects pressed a button using their right index finger. Because monkeys heard the opening sound of solenoid valve whenever they obtained reward during experiments, we recorded the sound in a sound clip and replayed it through headphone for every saccade to the 4th or later target which was generated within the specific time window. In the predictive condition, the window was ±120 ms (20% SOA) from the target onset, while in the remaining conditions, it was between 100 and 480 ms after target onset (i.e., 20% SOA before the next stimulus). Each condition was presented in two blocks of 25 trials, and blocks of different condition were presented sequentially (150 total trials for each subject). The block order was counterbalanced across subjects.

### Data acquisition and analysis

Eye movement data were digitized and sampled at 1 kHz, and were saved in files along with event timestamp during experiments. Data were analysed offline using Matlab (Mathworks). For the quantitative analysis, mean saccade latencies, mean inter-saccadic interval (ISI), and coefficient of variation (CV) of saccade latencies and ISI were calculated for each condition. In each figure, the inter-trial variability of these values was expressed by ±SD. For each animal and condition, we obtained the data from 5 experimental sessions. On average, each session contained 485 ± 135 trials (SD, *n* = 30, ranged from 258–932 trials). Because the variations between sessions were small (e.g., see Supplementary Fig. S2), we reported the means and SDs of individual trials that were pooled across sessions in most figures. A two-way analysis of variance (ANOVA) for the means of different experiments was used to evaluate factors affecting saccade latency, ISI, and CV. Post hoc multiple comparisons (t-test with Bonferroni correction) were also conducted as necessary. To examine the individual difference in monkeys, we also conducted ANOVAs including the subject factor. To evaluate the effects of target sequence on saccade timing, we adopted circular statistics by converting saccade latency into the phase data, where the times of right and left target onset corresponded to 0° and 180°, respectively (Fig. 2b and Supplementary Fig. S1). The effect of target sequence on saccade timing was assessed using a Watson-Williams test, which detected a difference between vectors generated from two circular distributions^27^. Details of statistical tests are provided in the relevant text in the Results. To examine how the animals maintained internal rhythm during the error-clamp condition, correlation coefficients between consecutive ISI were computed (Supplementary Fig. S4b). In addition, the variations of saccade timing relative to the start of error-clamp manipulation were compared with the simulation results of the “single-interval model” (Supplementary Fig. S4c). For this simulation, the ISIs at a given sequence position were shuffled across different trials for 1000 times for each monkey to estimate the variation based on the assumption that every saccade timing was determined independently.

## Electronic supplementary material

Supplementary Figures S1-4
