# Supplementary material for: Predictive and tempo-flexible synchronization to a visual metronome in monkeys
Source: Sci Rep. 2017 Jul 21;7:6127. doi: 10.1038/s41598-017-06417-3 (PMC5522449; doi:10.1038/s41598-017-06417-3)
Supplement: Supplementary file 1 — Supplementary Figures S1-4 [file 41598_2017_6417_MOESM1_ESM.pdf]

Supplementary Information

"Predictive and tempo-flexible synchronization to a visual metronome in monkeys"

by Ryuji Takeya, Masashi Kameda, Aniruddh D. Patel & Masaki Tanaka

Supplementary Figures S1–4

Supplementary Figure S1 for Takeya et al.

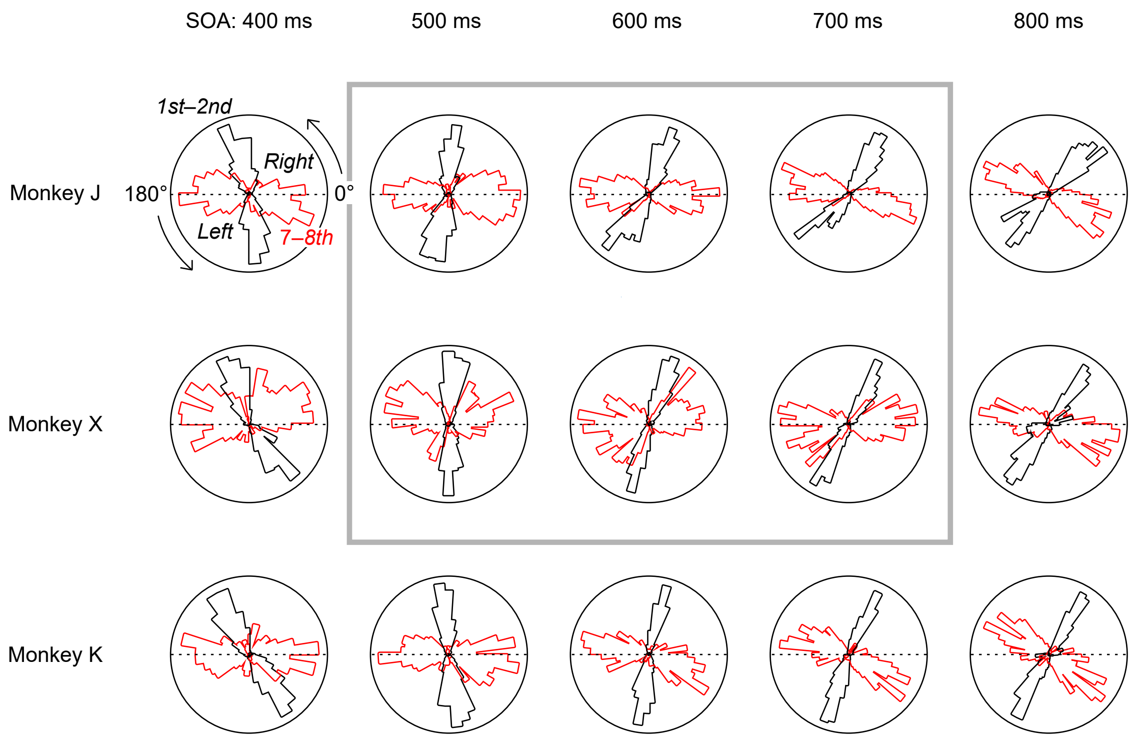

Supplementary Figure S1 | Circular histograms of latencies for the 1st–2nd (black) and 7–8th (red) saccades in the sequence with different SOAs. Note that monkeys J and X were not trained with SOAs of 500–700 ms before the experimental sessions (grey rectangle). For all SOA conditions and monkeys, the latency of the 1st–2nd saccades significantly differed from that of the 7–8th saccades (Watson-Williams test, *ps* $< 10^{-8}$).

Supplementary Figure S2 for Takeya et al.

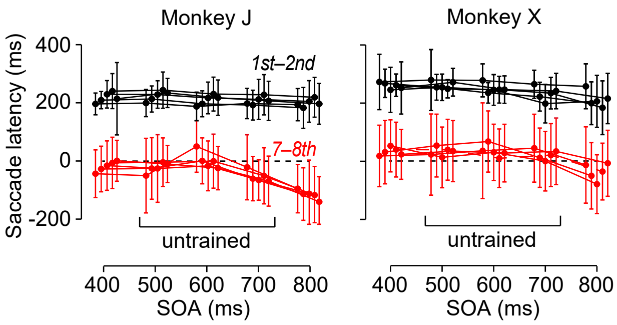

Supplementary Figure S2 | Synchronized saccades for the untrained SOAs during the five test sessions. Means (± SDs) of saccade latencies for different SOAs are plotted for individual sessions. Data from the same session are connected with lines and are shifted horizontally in the session order. Data from these five sessions are summarized in Figs. 3b and c. Note that both animals generated predictive saccades for the novel SOAs (500–700 ms) even during the 1st experimental session.

Supplementary Figure S3 for Takeya et al.

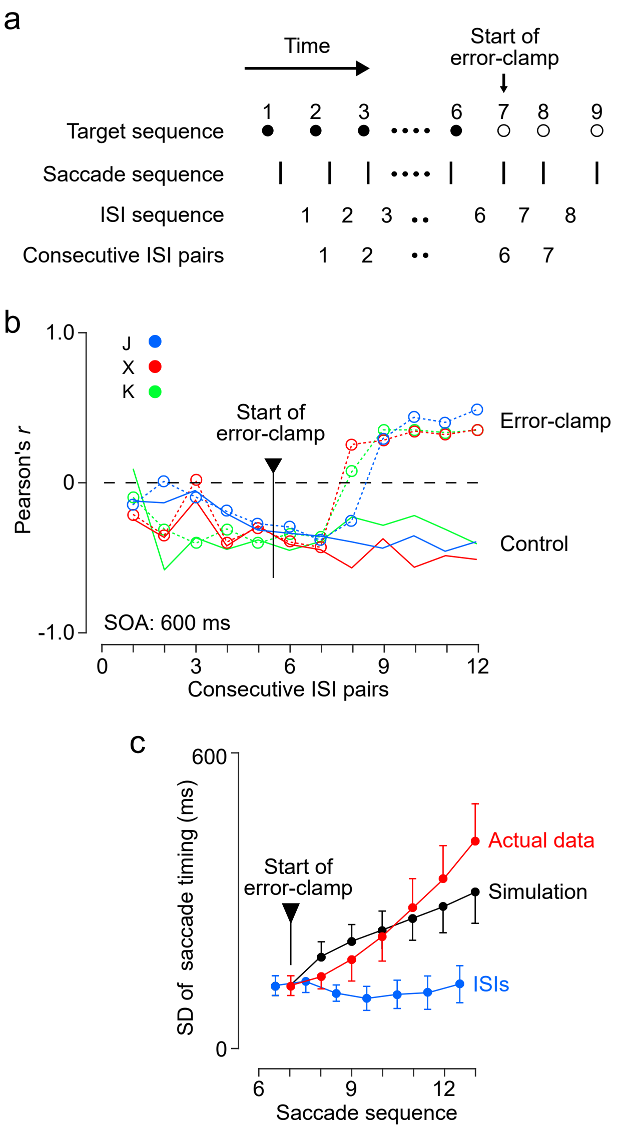

Supplementary Figure S3 | Time-series analysis of the variation of saccade timing in the error-clamp condition. (a) Definition of saccade sequence, inter-saccadic interval (ISI) sequence, and the sequence of consecutive ISI pairs. (b) Pearson's correlation coefficient of consecutive ISIs as a function of saccade sequence in the 600-ms SOA trials. Note that the correlation coefficient consistently remained negative in the control trials, whereas it rapidly increased to a positive value after the first few cycles of the error-clamp manipulation. (c) Variation (SD) of saccade timing during the error-clamp period. Red data points indicate the actual data obtained from three monkeys, representing SDs of saccade timing relative to the 6th saccade in the sequence (i.e., just before the start of error-clamp manipulation). Black data points plot the simulation results, showing the estimate of variance based on the assumption that each saccade timing was determined independently. Blue data points indicate SDs of ISIs for different saccades in the sequence. Error bars indicates SD of three monkeys.

Supplementary Figure S4 for Takeya et al.

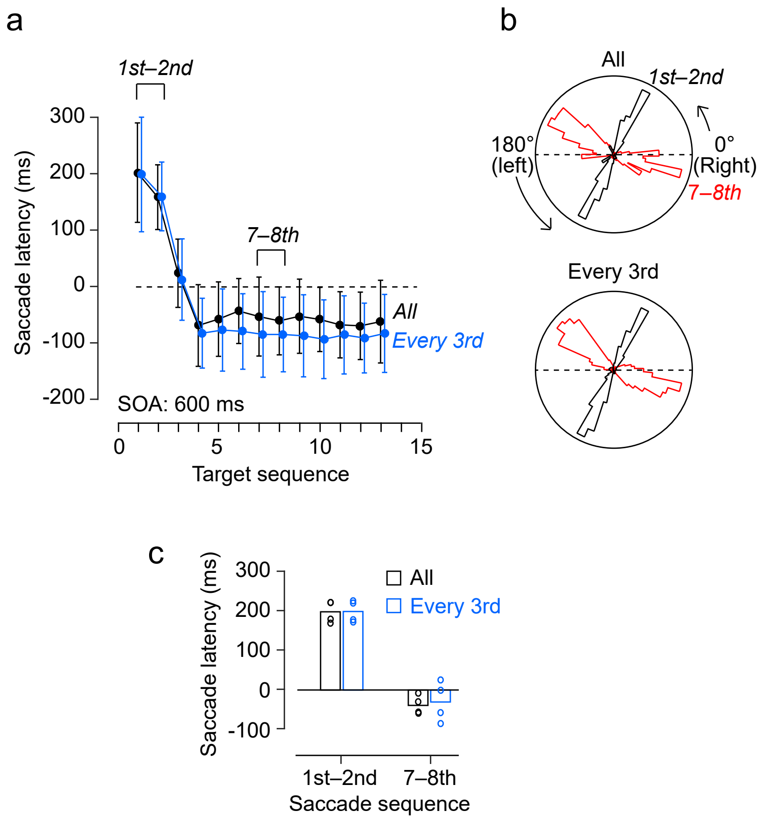

Supplementary Figure S4 | Effects of reward schedule. (a) In half of the trials, a liquid reward was delivered for every predictive saccade in the sequence (black), while it was given for every third saccade in the other half of the trials (blue). The amount of single reward in the latter condition was three times greater that the former condition so that the total amount of reward in each trial was equalized. (b) Circular histograms of the 1st–2nd (black) and the 7–8th (red) saccade latencies in the sequence for the data shown in a. (c) Summary of the data from 4 monkeys. Two of them participated in the other experiments (monkeys K and J), while the remaining animals (W and I) were only used for this particular experiment. A two-way ANOVA revealed a significant effect of saccade sequence ($F_{(1, 12)} = 184.0$, $p < 10^{-7}$) but showed no effect of reward schedule ($F_{(1, 12)} = 0.08$, $p = 0.79$) or interaction ($F_{(1, 12)} = 0.05$, $p = 0.83$).
